# Efficacy and Safety of Adding Clopidogrel to Aspirin on Stroke Prevention among High Vascular Risk Patients: A Meta-Analysis of Randomized Controlled Trials

**DOI:** 10.1371/journal.pone.0104402

**Published:** 2014-08-11

**Authors:** Shuying Chen, Qingyu Shen, Yamei Tang, Lei He, Yi Li, Hui Li, Mei Li, Ying Peng

**Affiliations:** 1 Department of Neurology, Sun Yat-sen Memorial Hospital, Sun Yat-Sen University, Guangzhou, Guangdong Province, China; 2 Department of Neurology, Affiliated Boji Hospital, Sun Yat-Sen University, Guangzhou, Guangdong Province, China; 3 Department of Neurology, Guangdong General Hospital, Guangzhou, Guangdong Province, China; Kaohsiung Chang Gung Memorial Hospital, Taiwan

## Abstract

**Objectives:**

Whether clopidogrel should be added to aspirin for stroke prevention remained controversial for the risk of hemorrhagic complications. This meta-analysis was aimed to assess the efficacy and safety of adding clopidogrel to aspirin on stroke prevention in high vascular risk patients, and to provide evidence for a suitable duration of dual antiplatelet therapy.

**Methods:**

We searched PubMed, EMBase, OVID and *Cochrane Central Register of Controlled Trials* (up to June, 2013) for randomized controlled trials evaluating the efficacy and safety of clopidogrel plus aspirin versus aspirin alone in high vascular risk patients. Comparisons of stroke and hemorrhagic complications between treatment groups were expressed by the pooled Relative Risks (RRs) with 95% Confidence Intervals (CIs).

**Results:**

Fifteen trials with a total of 97692 intention-to-treat participants were included with duration of follow-up ranging from 7 days to 3.6 years. Dual antiplatelet therapy reduced all stroke by 21% (RR: 0.79, 95% CI: 0.73–0.85) with no evidence of heterogeneity across the trials (*P* = 0.27, *I* ^2^ = 17%).The effects were consistent between short-term subgroup (≤1 month, RR: 0.76, 95% CI: 0.67–0.85) and long-term subgroup (≥3 months, RR: 0.81, 95% CI: 0.73–0.89). The risk of major bleeding was not significantly increased by dual antiplatelet therapy in short-term subgroup (RR: 1.11, 95% CI: 0.91–1.36), while significantly increased in long-term subgroup (RR: 1.52, 95% CI: 1.36–1.69). Long-term dual antiplatelet therapy substantially increased the risk of intracranial bleeding (RR: 1.76, 95% CI: 1.22–2.54).

**Conclusions:**

This meta-analysis demonstrates that short-term combination of clopidogrel and aspirin is effective and safe for stroke prevention in high vascular risk patients. Long-term combination therapy substantially increases the risk of major bleeding and intracranial bleeding.

## Introduction

Antiplatelet therapy has been recommended as the standard practice for stroke prevention in high vascular risk patients. Aspirin or clopidogrel monotherapy has been considered safe but not effective enough in these patients. Over the past decade, several large-scale clinical trials [1], [2], [3], [4], [5], [6], [7] have studied the efficacy and safety of adding clopidogrel to aspirin (dual antiplatelet therapy) on the prevention of cerebrovascular events and other ischemic events, but have resulted in conflicting directions, especially on the safety evaluation. Previous meta-analysis [8] including all vascular risk patients has concluded that dual antiplatelet therapy gets more risk reduction in stroke, but significantly increases the risk of major bleeding, compared with aspirin alone. Therefore, physicians have still been hesitating to give dual antiplatelet therapy to part of high vascular risk patients.

However, we observed that there was clinical heterogeneity on the treatment duration of dual antiplatelets across the relevant trials. Trials with long-term (≥3 months) dual antiplatelet therapy [1], [3], [6], [7] tended to result in higher risk of hemorrhagic complications than those with short-term (≤1 month) dual antiplatelet therapy [4], [5]. The newly published Clopidogrel in High-risk patients with Acute Nondisabling Cerebrovascular Events (CHANCE) trial also demonstrated that dual antiplatelet therapy for 21 days followed by clopidogrel for 3 months was safe and more effective than aspirin alone in preventing recurrence of stroke. We hypothesized that treatment duration of dual antiplatelets would have effect on the risk of hemorrhagic complications and one-month treatment would provide effective prevention on stroke and guarantee the safety.

Therefore, we initiated this meta-analysis to assess the efficacy and safety of adding clopidogrel to aspirin on stroke prevention in high vascular risk patients. Based on our hypothesis, we would perform subgroup analysis on the treatment duration and try to provide evidence for a suitable duration of dual antiplatelet therapy for stroke prevention.

## Methods

### Search strategy

PRISMA (Preferred Reporting Items for Systematic reviews and Meta-Analysis) checklist was provided as Checklist S1. A detailed protocol (Text S1) was developed before conduct of this study, according to the PRISMA statement [9]. We searched the electronic databases including PubMed, EMBase, OVID and *Cochrane Central Register of Controlled Trials* (up to June, 2013) to identify studies comparing the combination of clopidogrel and aspirin with aspirin alone, restricted to English only. Keywords, PubMed MeSH and free texts search were combined with the following keywords: clopidogrel, aspirin, Plavix, dual antiplatelet therapy, monotherapy, stroke, hemorrhage, hemorrhagic, RCTs, randomized controlled trial. After removing duplicate reports and unrelated articles, reference lists of the remaining articles and previous related meta-analyses were scrutinized to reveal additional related articles.

### Inclusion and exclusion criteria for study selection

We included the studies if they met the following criteria: 1) randomized controlled trials (RCTs); 2) comparing the combination of clopidogrel and aspirin with aspirin alone; 3) reporting clinical outcomes of stroke or bleeding events. The report with the most completed data was used when more than one publication were generated from one study.

We excluded these studies: 1) single dose of the combination of clopidogrel and aspirin; 2) without details on our pre-specified outcomes for analysis; 3) retrospective studies, editorials, letters, review articles, case reports, and animal experimental studies.

### Data extraction and assessment of risk of bias

Data were extracted independently by 2 investigators (SY Chen, QY Shen). Discrepancies were resolved by consensus or a third author adjudication (YM Tang). Details of the following items were abstracted: 1) baseline characteristics of participants; 2) interventions and treatment duration in each group; 3) definitions of the pre-specified outcomes; 4) positive events of pre-specified outcomes and total numbers of participants in each group, duration of follow-up, loss of follow-up, intention-to-treat analysis.

According to the Cochrane collaboration's tool for assessing risk of bias, we assessed the risk of bias of the included RCTs with the following domains: generation of random sequence; allocation concealment; blinding of participants and personnel; blinding of outcome assessment; incomplete outcome data; selecting reporting; and other potential sources of bias.

### Outcomes

Our primary outcomes were all stroke (including both ischemic stroke and hemorrhagic stroke) and major bleeding. Our secondary outcomes were ischemic stroke, hemorrhagic stroke and intracranial bleeding. The diagnostic criteria for outcomes of stroke and intracranial bleeding were generally similar in the included trials and accepted in this meta-analysis. The grading criteria for bleeding events varied across the included trials. Major bleeding in this meta-analysis was mainly defined as moderate to severe extracranial bleeding requiring blood transfusion, or causing a decrease in hemoglobin level of ≥3 g/dl, as well as intracranial bleeding.

### Statistical analysis

Results of this meta-analysis were expressed as pooled Relative Risks (RRs) with 95% Confidence Intervals (CIs) for dichotomous outcomes. A value of *P*<0.05 was considered statistically significant. Heterogeneity across trials was assessed via a standard Chi square test with significance being set at *P*<0.10 and also assessed by means of *I* ^2^. An *I* ^2^ value>50% was defined as high heterogeneity. Fixed-effect model was used for statistical analysis when low heterogeneity was assessed. Random-effect model was used when there was high heterogeneity across the trials. As grading criteria of bleeding events varied across the included trials, random-effect model was applied for the endpoint of major bleeding, considering the heterogeneity. Sensitivity analysis was performed to measure the effect of included RCTs. Subgroup analysis was performed on the treatment duration of dual antiplatelets. The included trials were assigned to short-term (≤1 month) or long-term (≥3 months) subgroups. Subgroup analysis on primary disease of the included population was also performed underneath each treatment duration subgroup to test the potential different effects of dual antiplatelet therapy between patients with and without previous stroke or TIA. Meta-regression was performed to recognize the source of heterogeneity. Funnel plots were used to screen for potential publication bias. When the funnel plots presented asymmetric, the “trim and fill” method [10] was used to adjust the results. Statistical analysis was performed on Review Manager 5.2 (The Cochrane Collaboration, Oxford, England) and Stata 12.0 (StataCorp LP, USA).

## Results

### Study selection

**Figure 1:**
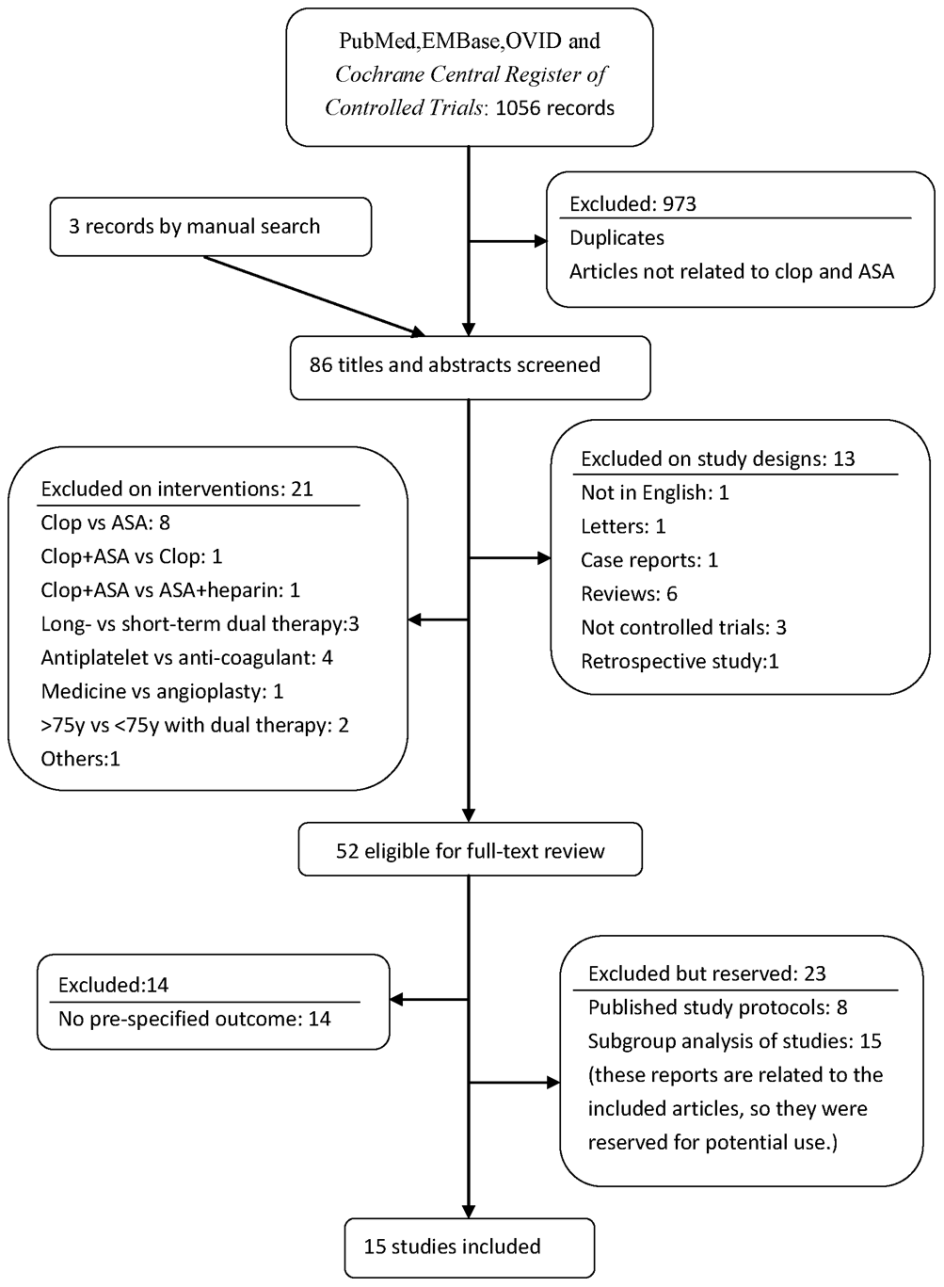
Flow diagram of study selection. Clop indicates clopidogrel and ASA indicates aspirin.

A total of 15 RCTs [1], [2], [3], [4], [5], [6], [7], [11], [12], [13], [14], [15], [16], [17], [18], [19] with 97692 intention-to-treat participants were identified for inclusion from 86 potentially relevant publications. Eight studies had publications of their rationales and designs [20], [21], [22], [23], [24], [25], [26], [27]. The details for exclusion of publications and the number of studies finally included in the review were showed in Figure 1, according to the PRISMA statement [9].

### Baseline characteristics and risk of bias assessment

The baseline characteristics of the included 15 trials were summarized in Table 1. The sample sizes of the included trials ranged from 79 to 45825. Mean ages of the participants ranged from 56 to 81 years and the percentages of females ranged from 10.6% to 57.5%. The included trials enrolled participants with previous cerebrovascular diseases [1], [2], [12], [16], [17], coronary arterial diseases [4], [5], [6], [18], multiple atherothrombotic risk factors [3], high risk of arterial thromboembolism [7], [11], or revascularization conditions [13], [14], [28]. The Clopidogrel for High Atherothrombotic Risk and Ischemic Stabilization, Management, and Avoidance (CHARISMA) trial [3] recruited patients with multiple atherothrombotic risk factors or established cardiovascular diseases, which included cerebrovascular diseases, coronary diseases and symptomatic peripheral arterial diseases. Medical history and high risk factors were described in most of the studies (Table S1). Six trials [4], [5], [12], [16], [17], [28] had dual antiplatelet therapy for less than 1 month, and the others for more than 3 months. The baseline characteristics were balanced between study arms in each trial.

**Table 1:** Baseline characteristics and design features of included trials.

Study	N of pts*		F, %		Age, y		Inclusion criteria	Prior stroke/TIA	Experimental group	Control group	Duration of Clop+ASA	Follow-up	
	Exp/Ctrl							N (%)				Duration	Lost
CARESS 2005[12]	51/56	31/30		66/63			Stroke/TIA(≤3 m) and carotid stenosis	107 (100)	Clop 300 →75 mg +ASA 75 mg	ASA 75 mg	7d	7d	None
CLAIR 2010[16]	46/52	22/23		59/56			Stroke/TIA(≤7d) and intracranial stenosis	98 (100)	Clop 300 →75 mg +ASA 75∼160 mg	ASA 75∼160 mg	7d	7d	1%
COMMIT 2005[4]	22961/22891	28/28		61/61			Acute MI with ST changes(≤24 hrs)	NR	Clop 75 mg+ASA 162 mg	ASA 162 mg	14.9d	15d (∼28d)	<1%
CHANCE 2013[17]	2584/2586	33/35		63/62			Acute minor stroke or TIA (≤24 hrs)	5170 (100)	Clop 300→75 mg ×90d +ASA 75 mg ×21d	ASA 75 mg×90d	21d	90d	0.7%
CLARITY 2005[5]	1752/1739	20/19		58/57			MI with ST elevation(≤12 hrs)	NR	Clop 300→75 mg +ASA 150∼325 →75∼162 mg	ASA 150∼325 →75∼162 mg	Median of 4 doses	30d	NR
Sun JC, 2010[28]	49/50	6/14		66/65			Post CABG	5 (5.0)	Clop 300→75 mg +ASA 325→81 mg	ASA 325→81 mg	30d	30d	None
FASTER 2007[2]	198/194	43/52		68/68			TIA or minor stroke(≤24 hrs)	392 (100)	Clop 300→75 mg +ASA 81 mg	ASA 81 mg	90d	90d	1.8%
Ussia GP 2011[11]	40/39	50/59		80/81			Transcatheter aortic valve implantation	10 (12.7)	Clop 300 →75 mg×3 m +ASA 100 mg	ASA 100 mg	3 m	6 m	None
CURE 2001[6]	6259/6303	39/38		64/64			ACS without ST elevation(≤24 hrs)	506 (4.0)	Clop 300→75 mg +ASA 75∼325 mg	ASA 75∼325 mg	9 m	3∼12 m	<1%
CASCADE 2010[13]	56/57	9/12		65/68			CABG	NR	Clop 75 mg+ASA 162 mg	ASA 162 mg	1y	1y	None
CASPAR 2010[14]	425/426	25/24		67/66		Vascular bypass grafting for PAD		NR	Clop 75 mg +ASA 75∼100 mg	ASA 75∼100 mg	351d	1y (6–24 m)	2.2%
REAL-LATE/ZEST-LATE 2010[18]	1357/1344	30/31		62/62		Stents used >12 m		102 (3.8)	Clop 75 mg+ASA 100∼200 mg	ASA 100∼200 mg	12.8 m	19.2 m	<1%
CHARISMA 2006[3]	7802/7801	30/30		64/64		Multiple athero- thrombotic risk factors, CAD, CVD or PAD		5701 (36.5) 4320 (27.7) ¶	Clop 75 mg +ASA 75∼162 mg	ASA 75∼162 mg	28 m	28 m	<0.5%
SPS3 2012[1]	1517/1503	38/36		63/63		Symptomatic lacunar stroke(≤180d)		3020 (100)	Clop 75 mg+ASA 325 mg	ASA 325 mg	3.5y	3.5y	2%
ACTIVE-A 2009[7]	3772/3782	41/42		71/71		AF, ≥1 risk factor for stroke§		992 (13.1)	Clop 75 mg+ASA 75∼100 mg	ASA 75∼100 mg	3.6y	3.6y	<1%

ACS: acute coronary syndrome; AF: atrial fibrillation; ASA: aspirin; CABG: coronary arterial bypass graft; CAD: coronary arterial disease; CVD: cerebrovascular disease; Clop: clopidogrel; Exp/Ctrl: data of the corresponding items in experimental group and control group, separately; F: female; MI: myocardial infarction; NR: not reported. TIA: transient ischemic attack. PAD: peripheral arterial disease.

*Number of patients.

¶ Documented cerebrovascular diseases during previous 5 years.

§ Risk factors for stroke: an age of 75 years or more; systemic hypertension during treatment; previous stroke, transient ischemic attack, or non–central nervous system systemic embolism; a left ventricular ejection fraction of less than 45%; peripheral vascular disease; or an age of 55 to 74 years and diabetes mellitus or coronary artery disease.

Risk of bias of individual trials was assessed according to the Cochrane collaboration's tool for assessing risk of bias (Table S2). Risk of bias summary and risk of bias graph (Figure S1–S2) showed that this meta-analysis was based mainly on studies with low risk of bias and would provide convincing evidence for clinical decision.

### Efficacy on stroke prevention

**Figure 2:**
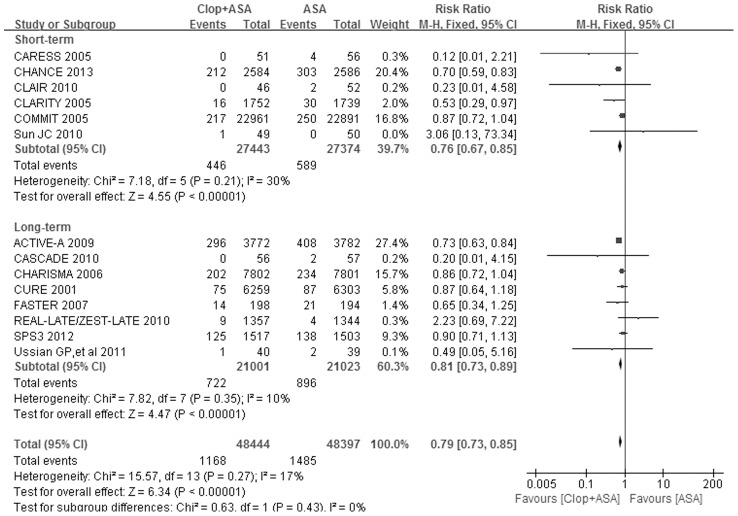
Forest plot of Clop+ASA vs. ASA on all stroke. ASA indicates aspirin; CI, confidence interval; Clop, clopidogrel; and M-H, Mantel-Haenszel method.

Fourteen trials including 96841 participants reported all stroke (both ischemic and hemorrhagic stroke) incidence. As shown in Table 2 and Figure 2, the pooled RR for all stroke by dual antiplatelet therapy versus (vs.) aspirin alone was 0.79 (95% CI: 0.73–0.85, *P*<0.00001) with no statistically significant evidence of heterogeneity across the trials (*I* ^2^ = 17%, *P* = 0.27). The pooled RRs for all stroke were 0.76 (95% CI: 0.67–0.85, *P*<0.00001) in short-term subgroup and 0.81 (95% CI: 0.73–0.89, *P*<0.00001) in long-term subgroup (Table 2 and Figure 2). The *P* value for interaction test between these two subgroups was 0.43 (Figure 2).

**Table 2:** Summarized results of meta-analysis.

Outcomes of interest	Studies number	Patients number		RR(95%CI)	*P*-value	Heterogeneity			
		Exp *	Ctrl #			Chi^2^	df	*P*-value	*I* ^2^,%
**Any stroke**	14	48444	48397	0.79 (0.73–0.85)	**<0.00001**	15.57	13	0.27	17
Short-term	6	27443	27374	0.76 (0.67–0.85)	**<0.00001**	7.18	5	0.21	30
Long-term	8	21001	21023	0.81(0.73–0.89)	**<0.00001**	7.82	7	0.35	10
**Ischemic stroke** ‡	10	45246	45225	0.76(0.70–0.82)	**<0.00001**	9.16	9	0.42	2
Short-term	4	25642	25585	0.74(0.65–0.85)	**<0.00001**	4.19	3	0.24	28
Long-term	6	19604	19640	0.77(0.69–0.85)	**<0.00001**	4.85	5	0.43	0
**Hemorrhagic stroke**	10	45246	45225	1.12(0.87–1.44)	0.38	4.61	6	0.59	0
Short-term	4	25642	25585	0.98(0.69–1.39)	0.92	0.00	1	0.97	0
Long-term	6	19604	19640	1.30(0.90–1.87)	0.16	3.54	4	0.47	0
**Major bleeding**	14	47493	47459	1.42(1.25–1.62)	**<0.00001**	15.57	11	0.16	29
Short-term	6	27424	27354	1.11(0.91–1.36)	0.30	0.81	3	0.85	0
Long-term	8	20069	20105	1.52(1.36–1.69)	**<0.00001**	7.50	7	0.38	7
**Intracranial bleeding**	7	30278	30197	1.25(0.98–1.61)	0.07	7.67	4	0.10	48
Short-term	4	24791	24718	0.92(0.66–1.30)	0.65	0.63	1	0.43	0
Long-term	3	5487	5479	1.76(1.22–2.54)	**0.002**	0.84	2	0.66	0

RR: relative risk; CI: confidence interval.

* Indicating clopidogrel plus aspirin group.

# Indicating aspirin plus placebo group.

¶ Degree of freedom.

‡ Ischemic stroke including stroke with uncertain causes.

The pooled RR for ischemic stroke (including stroke with uncertain causes) by dual antiplatelet therapy vs. aspirin monotherapy was 0.76 (95% CI: 0.70–0.82, *P*<0.00001, Table 2 and Figure S3). The effect was consistent between the short-term and long-term subgroups (interaction *P* = 0.71, Figure S3).

The pooled RR for hemorrhagic stroke by dual antiplatelet therapy vs. aspirin monotherapy was 1.12 (95% CI: 0.87–1.44, *P* = 0.38, Table 2 and Figure S4). Short-term dual antiplatelet therapy did not affect the risk of hemorrhagic stroke (RR: 0.98, 95% CI: 0.69–1.39, *P* = 0.92, Table 2 and Figure S4). Long-term dual antiplatelet therapy tended to increase the risk of hemorrhagic stroke but the difference had no statistical significance (RR: 1.30, 95% CI: 0.90–1.87, *P* = 0.16, Table 2 and Figure S4). The *P* value for interaction test between these two subgroups was 0.28 (Figure S4).

### Safety evaluation

**Figure 3:**
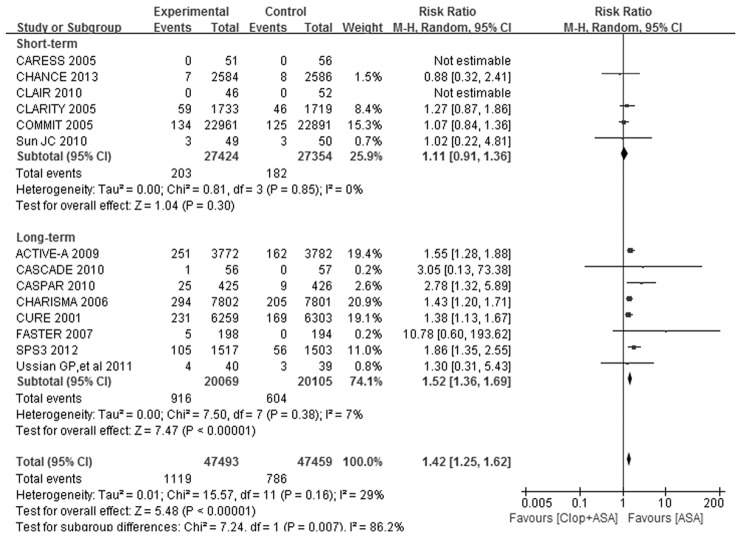
Forest plot of Clop+ASA vs. ASA on major bleeding. ASA indicates aspirin; CI, confidence interval; Clop, clopidogrel; and M-H, Mantel-Haenszel method.

As shown in Table 2 and Figure 3, the pooled RR for major bleeding by dual antiplatelet therapy vs. aspirin monotherapy was 1.42 (95% CI: 1.25–1.62, *P*<0.00001) without significant evidence of heterogeneity (*I* ^2^ = 29%, *P* = 0.16) across the trials. The risk of major bleeding were not significantly increased by dual antiplatelet therapy (RR: 1.11, 95% CI: 0.91–1.36, *P* = 0.30, Table 2 and Figure 3) in short-term subgroup. However, the risk of major bleeding was significantly increased by dual antiplatelet therapy in long-term subgroup (RR: 1.52, 95% CI: 1.36–1.69, *P*<0.00001, Table 2 and Figure 3). The *P* value for interaction test between short-term and long-term subgroups was 0.007 (Figure 3).

**Figure 4:**
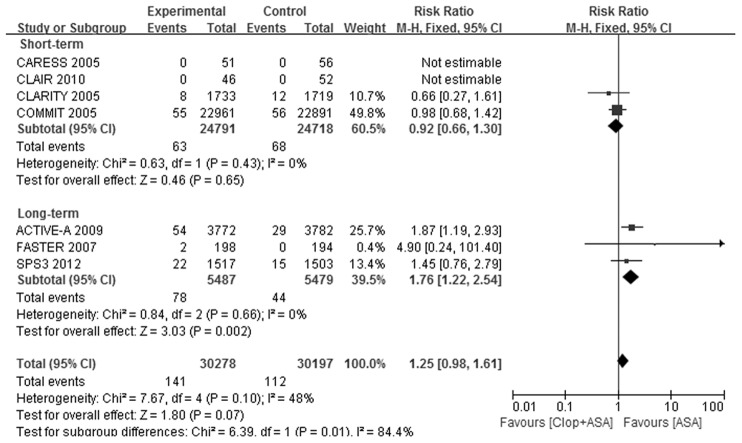
Forest plot of Clop+ASA vs. ASA on intracranial bleeding. ASA indicates aspirin; CI, confidence interval; Clop, clopidogrel; and M-H, Mantel-Haenszel method.

Only 7 trials [1], [2], [4], [5], [7], [12], [16] reported the incidence of intracranial bleeding. Intracranial bleeding was significantly increased by dual antiplatelet therapy (RR: 1.25, 95% CI: 0.98–1.61, Table 2 and Figure 4). Short-term dual antiplatelet therapy did not increase the risk of intracranial bleeding (RR: 0.92, 95% CI: 0.66–1.30), while long-term treatment substantially increased the risk of intracranial bleeding (RR: 1.76, 95% CI: 1.22–2.54, Table 2 and Figure 4), compared with aspirin alone. The interaction test *P* value between these two subgroups was 0.01(Figure 4).

### Subgroup analysis on primary disease

The effects of dual antiplatelet therapy on the outcomes of stroke (i.e., all stroke, ischemic stroke and hemorrhagic stroke) were consistent between patients with previous stroke or TIA and those with other vascular events or risk factors (Table 3 and Figure S5–10), irrespective of treatment duration.

**Table 3:** Subgroup analysis on primary diseases.

	Short-term treatment		Long-term treatment	
	RR (95% CI)	*P* for interaction	RR (95% CI)	*P* for interaction
All stroke				
Prior stroke/TIA	0.69 (0.58–0.81)	0.12	0.84 (0.71–0.99)	0.58
Other vascular event/risk	0.83 (0.70–0.99)		0.79 (0.70–0.89)	
Ischemic stroke				
Prior stroke/TIA	0.68 (0.57–0.81)	0.12	0.78 (0.66–0.93)	0.78
Other vascular event/risk	0.84 (0.69–1.04)		0.76 (0.67–0.86)	
Hemorrhagic stroke				
Prior stroke/TIA	1.00 (0.38–2.66)	0.97	1.56 (0.86–2.84)	0.43
Other vascular event/risk	0.98 (0.69–1.39)		1.15 (0.73–1.84)	
Major bleeding				
Prior stroke/TIA	0.88 (0.32–2.41)	0.64	1.70 (1.31–2.22)	0.31
Other vascular event/risk	1.12 (0.92–1.37)		1.47 (1.31–1.64)	
Intracranial bleeding				
Prior stroke/TIA	NA	NA	1.56 (0.83–2.95)	0.66
Other vascular event/risk	0.92 (0.66–1.30)		1.87 (1.19–2.93)	

CI: confidence interval; NA: not available; RR; relative risk; TIA: transient ischemic attack.

In long-term treatment trials, dual antiplatelet therapy increased the risk of major bleeding in both patients with previous stroke or TIA and those with other vascular events or risk factors (interaction *P* = 0.31, Table 3 and Figure S11). In short-term treatment trials, the risk of major bleeding was not significantly increased by by dual antiplatelete therapy in both trial cohorts (interaction *P* = 0.64, Table 3 and Figure S12). Long-term dual antiplatelet therapy did not show significantly different effect on intracranial bleeding between the two trial cohorts (interaction *P* = 0.66, Table 3 and Figure S13).

### Publication bias

Funnel plot analysis on the outcome of all stroke did not indicate significant publication bias (Figure S14). Funnel plot analysis on the outcome of major bleeding (Figure S15) presented asymmetrical and absence of trials at the left bottom of the plots. The conclusion for major bleeding was not changed and the funnel plot presented symmetrical after adjustment for publication bias by the “trim and fill” method (Figure S16).

## Discussion

As stroke could lead to disability and bring heavy burden to the family and the society, adding clopidogrel to aspirin for stroke prevention could be valuable clinical practice. However, the increased risk of bleeding should be taken into account. Previous clinical trials with long-term dual antiplatelet therapy tended to get risk increase in bleeding events [1], [3], [6], [7], though trials had dual antiplatelet therapy for less than 1 month tended to have nonsignificant increase in the risk of bleeding events [4], [5]. The results of the CHANCE trial [17] corresponded to our hypothesis.

However, the recently emerging systemic reviews and meta-analyses [8], [29], [30], [31], [32] have never focused on the treatment duration of dual antiplatelets. Santiago et al. [8] reported adding clopidogrel to aspirin had substantial relative risk reduction in stroke incidence, but increased the risk of major bleeding. Zhou et al. [30] also reported a relative risk increase in major bleeding and a small relative risk reduction in major cardiovascular events by adding clopidogrel to aspirin. Another systemic review and meta-analysis by Gouya et al [31] resulted in effective risk reduction in stroke and ischemic stroke without risk increase in intracranial bleeding among patients with vascular diseases. But they haven't considered the outcome of major bleeding. Wong et al [32] reported that dual antiplatelet therapy effectively prevented recurrent stroke without increasing the risk of major bleeding in patients with acute ischemic stroke and TIA. But they mainly included subjects with 21 days of dual antiplatelet therapy from the CHANCE trial and should not support for long-term dual antiplatelet therapy. Lee M et al [33] studied dual antiplatelet therapy lasting more than 1 year, but their results included effects of aspirin plus dipyridamole. Therefore, our analysis based on treatment duration of aspirin plus clopidogrel would be necessary and valuable for clinical decision.

In view of the overall effect, adding clopidogrel to aspirin significantly reduced all stroke incidence by 21% (Table 2 and Figure 2), and mainly prevented the occurrence of ischemic stroke (Table 2 and Figure S3). Hemorrhagic stroke was not significantly increased by dual antiplatelet therapy (Table 2 and Figure S4).Dual antiplatelet therapy significantly increased the risk of major bleeding by 42% (Table 2 and Figure 3), and tended to increase the risk of intracranial bleeding (*P* = 0.07, Table 2 and Figure 4).

In subgroup of short-term treatment, dual antiplatelet therapy substantially reduced the risk of all stroke and ischemic stroke, without significantly increasing the risk of hemorrhagic stroke, major bleeding or intracranial bleeding, compared with aspirin alone. In subgroup of long-term treatment, dual antiplatelet therapy also got risk reduction in all stroke and ischemic stroke, but evidently increased the risk of major bleeding and intracranial bleeding. The effects of dual antiplatelet therapy on stroke outcomes were consistent between short-term and long-term subgroups. However, there were differences between the two subgroups on the safety outcomes. The 95% CIs of RRs for major bleeding in short-term and long-term subgroups did not overlap with each other and there was evident heterogeneity of effect between the subgroups (interaction *P* = 0.007, Table 2 and Figure 3). That is to say, long-term treatment got substantially higher RR for major bleeding by dual vs. monotherapy than short-term treatment, while dual antiplatelet therapy did not increase the risk of major bleeding in short-term subgroup. The situation was similar to the outcome of intracranial bleeding (Table 2 and Figure 4). These results confirmed our hypothesis.

This meta-analysis included trial cohorts with different primary vascular diseases. Patients with previous cerebrovascular diseases have been considered to be in higher risk of stroke and intracranial bleeding than those without previous cerebrovascular diseases. However, patients with other vascular events (i.e., myocardial infarction and symptomatic peripheral arterial disease) would also get risk reduction in stroke by dual antiplatelet therapy, as they shared multiple common risk factors with patients with previous cerebrovascular diseases. As Santiago et al. [8] reported, the effect of dual antiplatelet therapy on stroke prevention was consistent across different trial cohorts. Our further subgroup analyses on primary diseases of the included population had similar conclusions (Table 3). The preventive effects on all stroke and ischemic stroke by dual antiplatelet therapy were consistent between patients with previous stroke or TIA and those with other vascular events or risk factors (Table 3). In subgroup analyses of short-term treatment trials, patients with prior stroke or TIA seemed to get more risk reduction in all stroke and ischemic stroke than those with other vascular events or risk factors, but the differences were not significant (interaction *P* = 0.12 for both outcomes) (Table 3, Figure S5 and Figure S7). The CHANCE trial [17] accounted for the most of participants in the subgroup of prior stroke or TIA, which might suggest that patients with acute minor stroke or TIA would get more benefits from short-term dual antiplatelet therapy. Subgroup analyses on primary diseases also showed the effects of dual antiplatelet therapy on major bleeding and intracranial bleeding was consistent between patients with previous stroke or TIA and those with other vascular events or risk factors. There was no evidence that patients with prior stroke or TIA would get significantly higher RR for hemorrhagic stroke or intracranial bleeding by dual vs. monotherapy than those with other vascular events or risk factors.

In addition, there were some participants in high risk of severe or even fatal conditions (i.e., post percutaneous coronary intervention) that dual antiplatelet therapy was strongly recommended for long-term treatment. The risk of fatal conditions should be taken into account and the risk of bleeding events should be assessed when determining the treatment duration of dual antiplatelets for these patients.

There were some limitations with our research. Firstly, only published data were included, which may cause potential publication bias due to the preferential publication of positive findings. Secondly, subgroup data were not always available in the included trials, which limited the capacity to fully explore effects in subgroups. Thirdly, different grading criteria of bleeding events were adopted in the included trials, as well as self-defined major bleeding. This may resulted in potential heterogeneity and affected the stringency on safety evaluation.

## Conclusions

In summary, combination of clopidogrel and aspirin for less than 1 month is effective and safe for stroke prevention in high vascular risk patients. Combination of clopidogrel and aspirin for more than 3 months substantially increases the risk of major bleeding and intracranial bleeding.

## Supporting Information

Figure S1 **Risk of bias summary.** Green indicates lower risk; yellow indicates unclear risk; red indicates high risk.
(TIF)

Figure S2 **Risk of bias graph.** Green indicates lower risk; yellow indicates unclear risk; red indicates high risk.
(TIF)

Figure S3 **Forest plot of Clop+ASA vs. ASA on ischemic stroke and uncertain causes.** ASA indicates aspirin; CI, confidence interval; Clop, clopidogrel; and M-H, Mantel-Haenszel method.
(TIF)

Figure S4 **Forest plot of Clop+ASA vs. ASA on hemorrhagic stroke.** ASA indicates aspirin; CI, confidence interval; Clop, clopidogrel; and M-H, Mantel-Haenszel method.
(TIF)

Figure S5 **Forest plot of Clop+ASA vs. ASA on all stroke with short-term treatment.** ASA indicates aspirin; CI, confidence interval; Clop, clopidogrel; and M-H, Mantel-Haenszel method.
(TIF)

Figure S6 **Forest plot of Clop+ASA vs. ASA on all stroke with long-term treatment.** CHARISMA sub-1 included the subgroup population with documented cerebrovascular diseases during previous 5 years and CHARISMA sub-2 included the residual population in CHARISMA trial. ASA indicates aspirin; CI, confidence interval; Clop, clopidogrel; and M-H, Mantel-Haenszel method.
(TIF)

Figure S7 **Forest plot of Clop+ASA vs. ASA on ischemic stroke with short-term treatment.** ASA indicates aspirin; CI, confidence interval; Clop, clopidogrel; and M-H, Mantel-Haenszel method.
(TIF)

Figure S8 **Forest plot of Clop+ASA vs. ASA on ischemic stroke with long-term treatment.** CHARISMA sub-1 included the subgroup population with documented cerebrovascular diseases during previous 5 years and CHARISMA sub-2 included the residual population in CHARISMA trial. ASA indicates aspirin; CI, confidence interval; Clop, clopidogrel; and M-H, Mantel-Haenszel method.
(TIF)

Figure S9 **Forest plot of Clop+ASA vs. ASA on hemorrhagic stroke with short-term treatment.** ASA indicates aspirin; CI, confidence interval; Clop, clopidogrel; and M-H, Mantel-Haenszel method.
(TIF)

Figure S10 **Forest plot of Clop+ASA vs. ASA on hemorrhagic stroke with long-term treatment.** CHARISMA sub-1 included the subgroup population with documented cerebrovascular diseases during previous 5 years and CHARISMA sub-2 included the residual population in CHARISMA trial. ASA indicates aspirin; CI, confidence interval; Clop, clopidogrel; and M-H, Mantel-Haenszel method.
(TIF)

Figure S11 **Forest plot of Clop+ASA vs. ASA on major bleeding with long-term treatment.** CHARISMA sub-1 included the subgroup population with documented cerebrovascular diseases during previous 5 years and CHARISMA sub-2 included the residual population in CHARISMA trial. ASA indicates aspirin; CI, confidence interval; Clop, clopidogrel; and M-H, Mantel-Haenszel method.
(TIF)

Figure S12 **Forest plot of Clop+ASA vs. ASA on major bleeding with short-term treatment.** ASA indicates aspirin; CI, confidence interval; Clop, clopidogrel; and M-H, Mantel-Haenszel method.
(TIF)

Figure S13 **Forest plot of Clop+ASA vs. ASA on intracranial bleeding with long-term treatment.** ASA indicates aspirin; CI, confidence interval; Clop, clopidogrel; and M-H, Mantel-Haenszel method.
(TIF)

Figure S14 **Funnel plot on the outcome of all stroke.** RR: relative risk; SE: standard error.
(TIF)

Figure S15 **Funnel plot on the outcome of major bleeding.** RR: relative risk; SE: standard error.
(TIF)

Figure S16 **Funnel plot on the outcome of major bleeding after adjustment of publication bias by the “trim and fill” method (by Stata 12.0).** RR: relative risk; SE: standard error.
(TIF)

Table S1 **Supplemental data for baseline characteristics.**
(DOC)

Table S2 **Risk of bias assessment in details.**
(DOC)

Text S1 **Study protocol.**
(DOC)

Checklist S1 **PRISMA 2009 Checklist.**
(DOC)
