# Supplementary figures and images for: Efficacy and Safety of Adding Clopidogrel to Aspirin on Stroke Prevention among High Vascular Risk Patients: A Meta-Analysis of Randomized Controlled Trials
Source: PLoS One. 2014 Aug 11;9(8):e104402. doi: 10.1371/journal.pone.0104402 (PMC4128803; doi:10.1371/journal.pone.0104402)

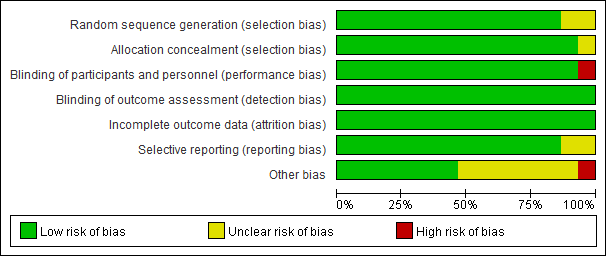

Supplement: Figure S1 — Risk of bias summary. Green indicates lower risk; yellow indicates unclear risk; red indicates high risk. (TIF) [file pone.0104402.s001.tif]

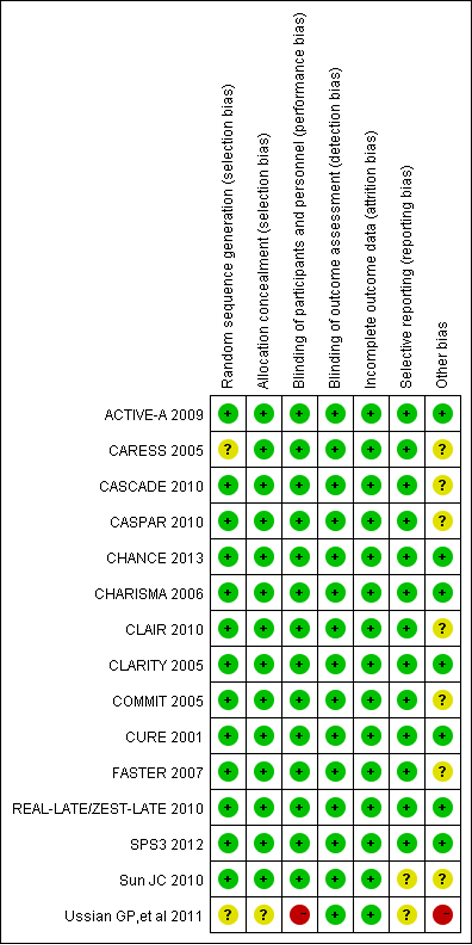

Supplement: Figure S2 — Risk of bias graph. Green indicates lower risk; yellow indicates unclear risk; red indicates high risk. (TIF) [file pone.0104402.s002.tif]

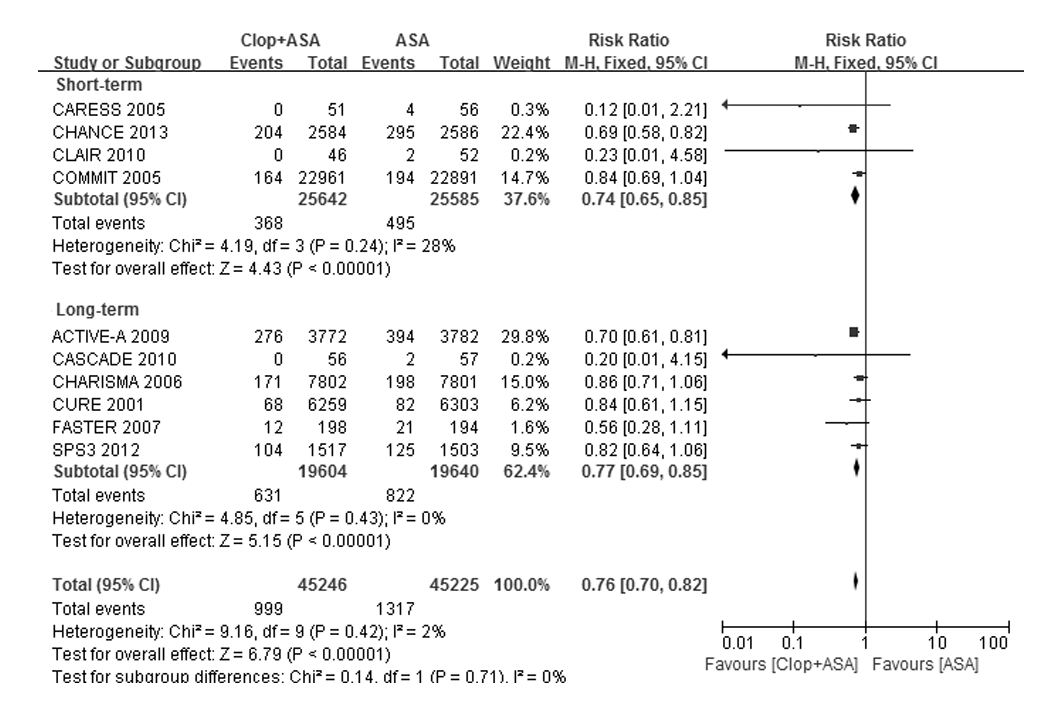

Supplement: Figure S3 — Forest plot of Clop+ASA vs. ASA on ischemic stroke and uncertain causes. ASA indicates aspirin; CI, confidence interval; Clop, clopidogrel; and M-H, Mantel-Haenszel method. (TIF) [file pone.0104402.s003.tif]

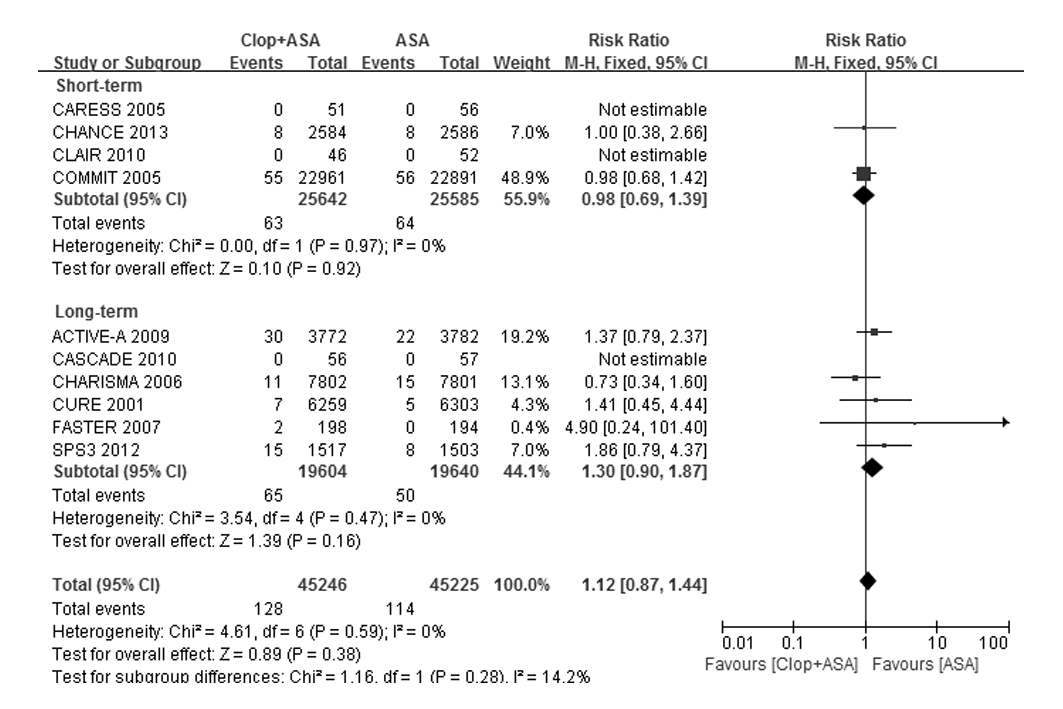

Supplement: Figure S4 — Forest plot of Clop+ASA vs. ASA on hemorrhagic stroke. ASA indicates aspirin; CI, confidence interval; Clop, clopidogrel; and M-H, Mantel-Haenszel method. (TIF) [file pone.0104402.s004.tif]

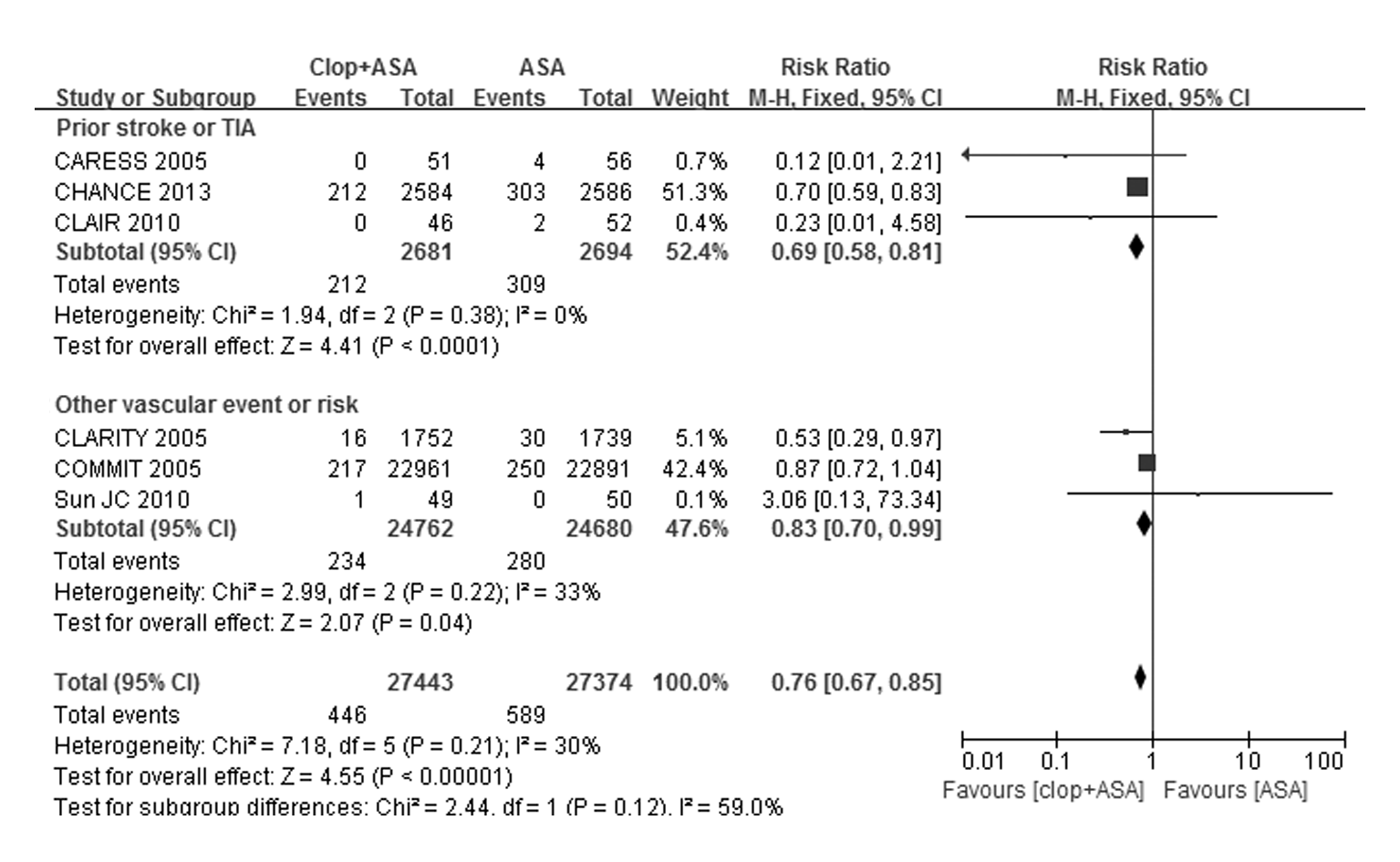

Supplement: Figure S5 — Forest plot of Clop+ASA vs. ASA on all stroke with short-term treatment. ASA indicates aspirin; CI, confidence interval; Clop, clopidogrel; and M-H, Mantel-Haenszel method. (TIF) [file pone.0104402.s005.tif]

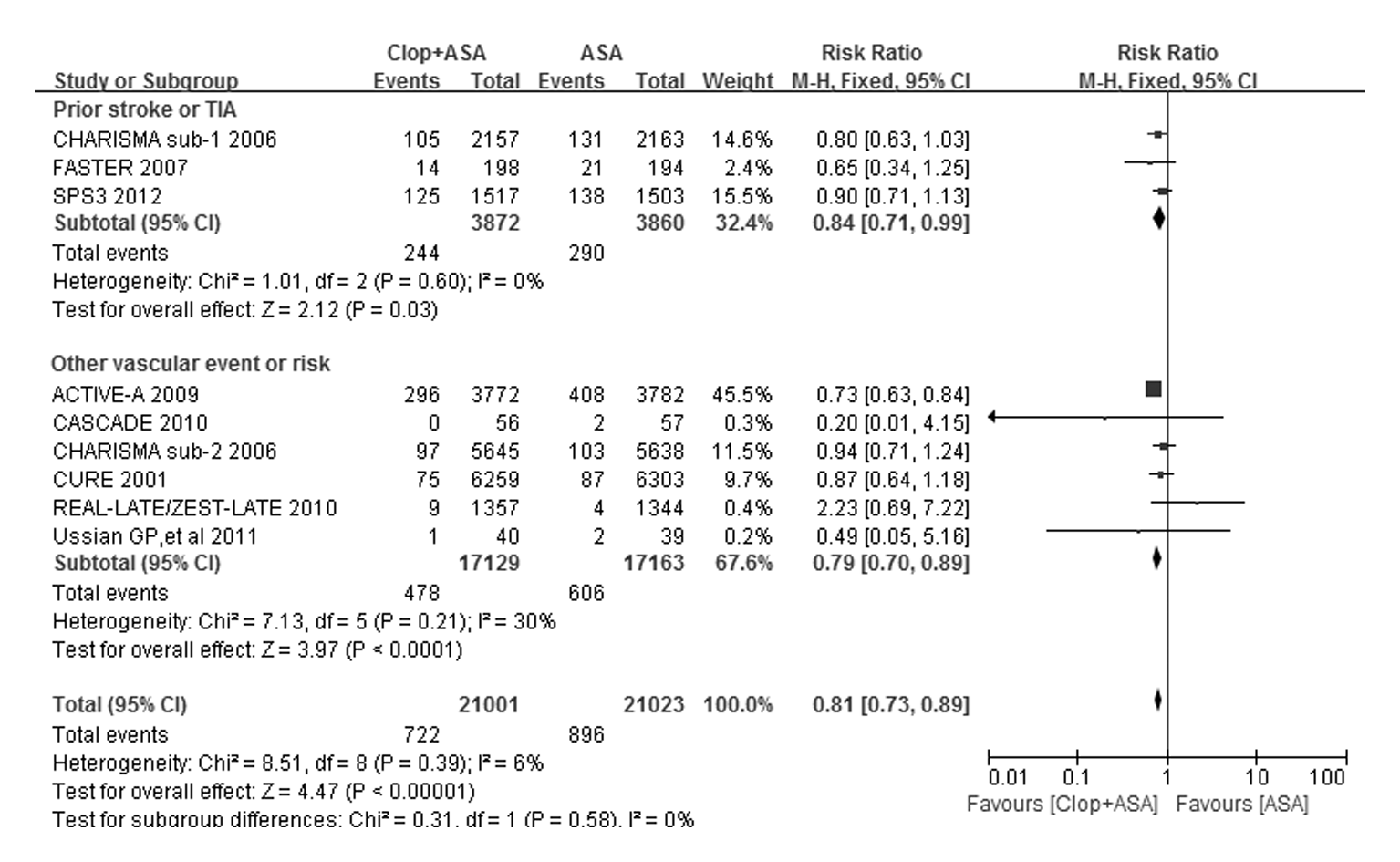

Supplement: Figure S6 — Forest plot of Clop+ASA vs. ASA on all stroke with long-term treatment. CHARISMA sub-1 included the subgroup population with documented cerebrovascular diseases during previous 5 years and CHARISMA sub-2 included the residual population in CHARISMA trial. ASA indicates aspirin; CI, confidence interval; Clop, clopidogrel; and M-H, Mantel-Haenszel method. (TIF) [file pone.0104402.s006.tif]

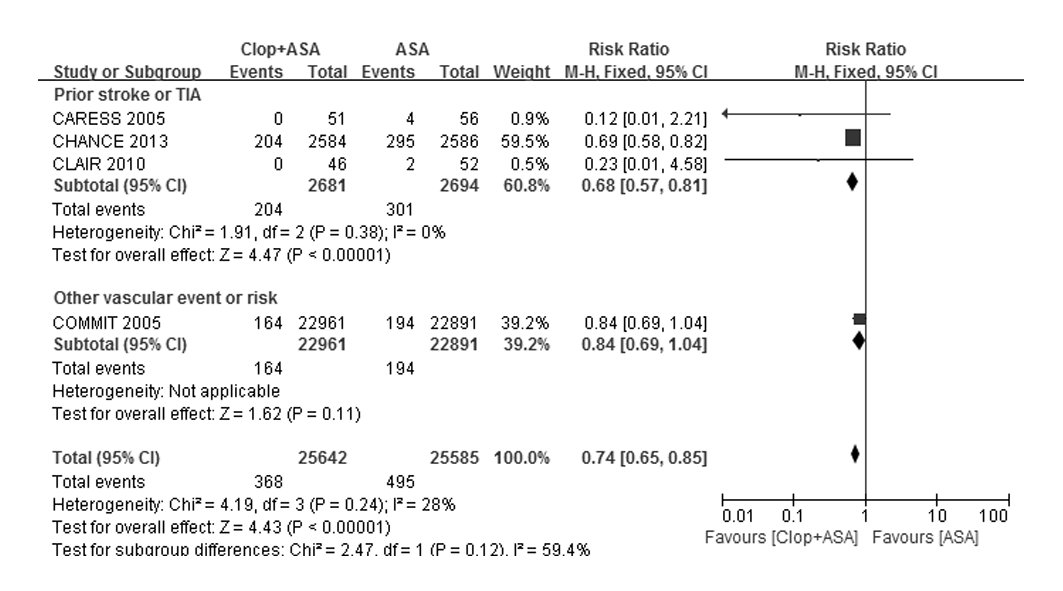

Supplement: Figure S7 — Forest plot of Clop+ASA vs. ASA on ischemic stroke with short-term treatment. ASA indicates aspirin; CI, confidence interval; Clop, clopidogrel; and M-H, Mantel-Haenszel method. (TIF) [file pone.0104402.s007.tif]

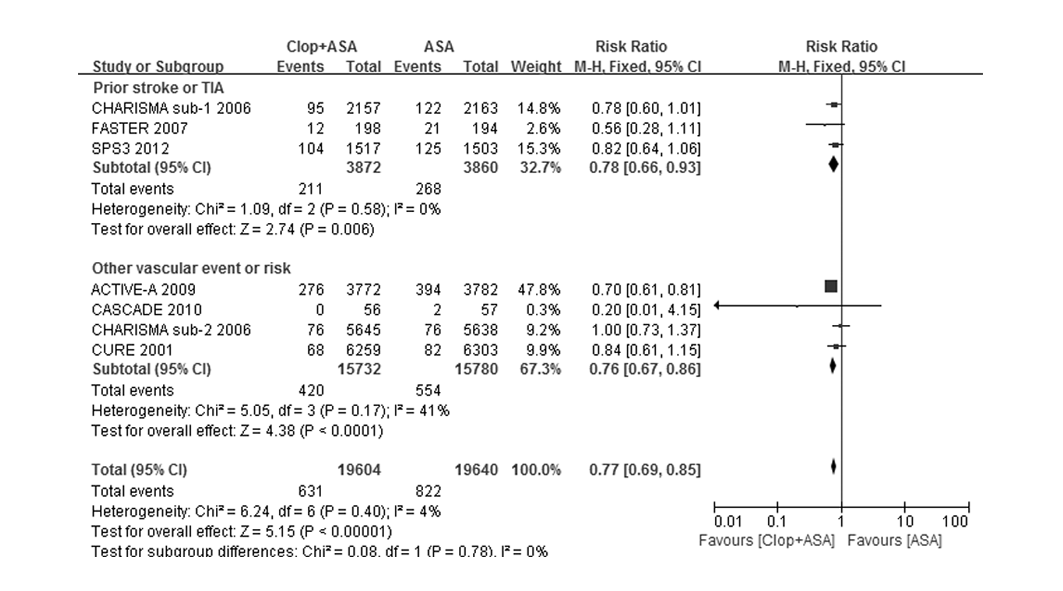

Supplement: Figure S8 — Forest plot of Clop+ASA vs. ASA on ischemic stroke with long-term treatment. CHARISMA sub-1 included the subgroup population with documented cerebrovascular diseases during previous 5 years and CHARISMA sub-2 included the residual population in CHARISMA trial. ASA indicates aspirin; CI, confidence interval; Clop, clopidogrel; and M-H, Mantel-Haenszel method. (TIF) [file pone.0104402.s008.tif]

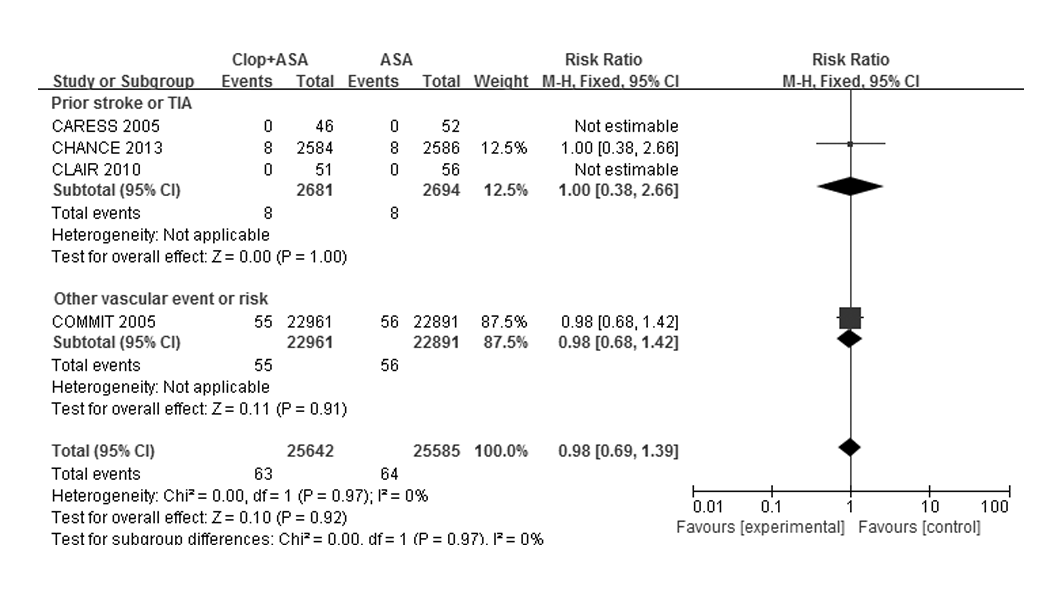

Supplement: Figure S9 — Forest plot of Clop+ASA vs. ASA on hemorrhagic stroke with short-term treatment. ASA indicates aspirin; CI, confidence interval; Clop, clopidogrel; and M-H, Mantel-Haenszel method. (TIF) [file pone.0104402.s009.tif]

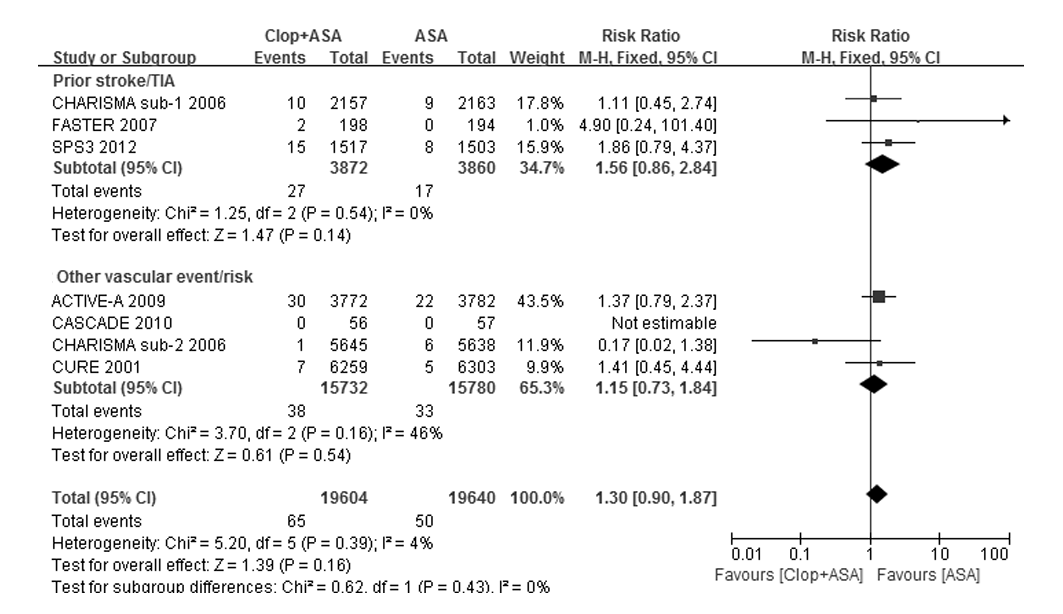

Supplement: Figure S10 — Forest plot of Clop+ASA vs. ASA on hemorrhagic stroke with long-term treatment. CHARISMA sub-1 included the subgroup population with documented cerebrovascular diseases during previous 5 years and CHARISMA sub-2 included the residual population in CHARISMA trial. ASA indicates aspirin; CI, confidence interval; Clop, clopidogrel; and M-H, Mantel-Haenszel method. (TIF) [file pone.0104402.s010.tif]

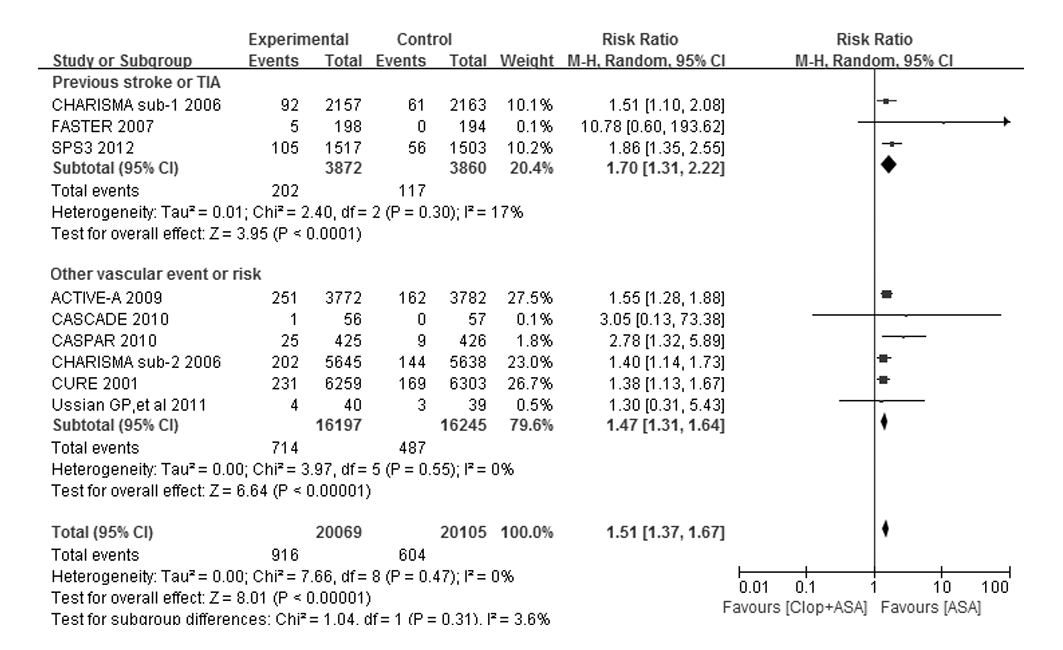

Supplement: Figure S11 — Forest plot of Clop+ASA vs. ASA on major bleeding with long-term treatment. CHARISMA sub-1 included the subgroup population with documented cerebrovascular diseases during previous 5 years and CHARISMA sub-2 included the residual population in CHARISMA trial. ASA indicates aspirin; CI, confidence interval; Clop, clopidogrel; and M-H, Mantel-Haenszel method. (TIF) [file pone.0104402.s011.tif]

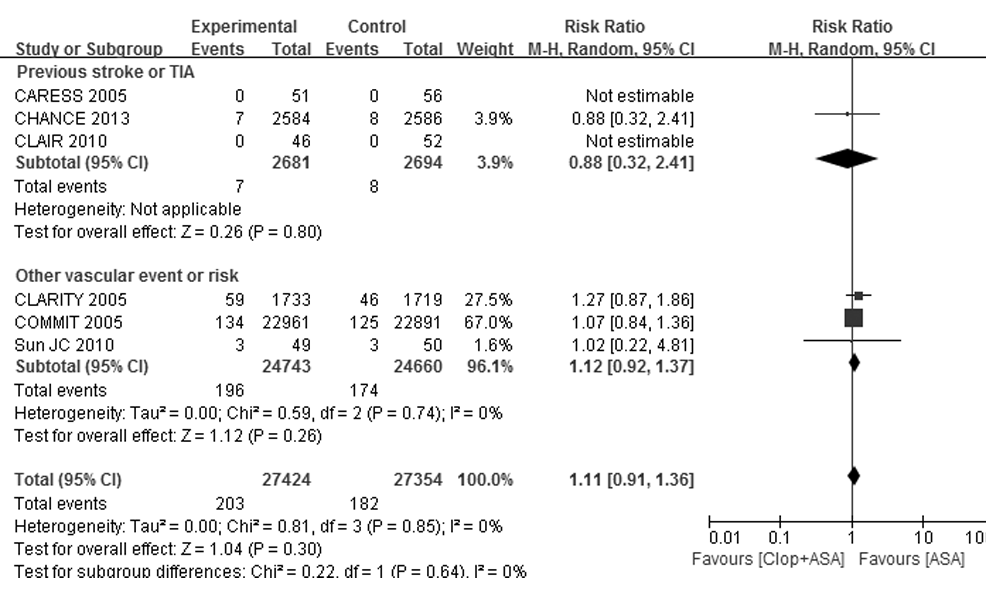

Supplement: Figure S12 — Forest plot of Clop+ASA vs. ASA on major bleeding with short-term treatment. ASA indicates aspirin; CI, confidence interval; Clop, clopidogrel; and M-H, Mantel-Haenszel method. (TIF) [file pone.0104402.s012.tif]

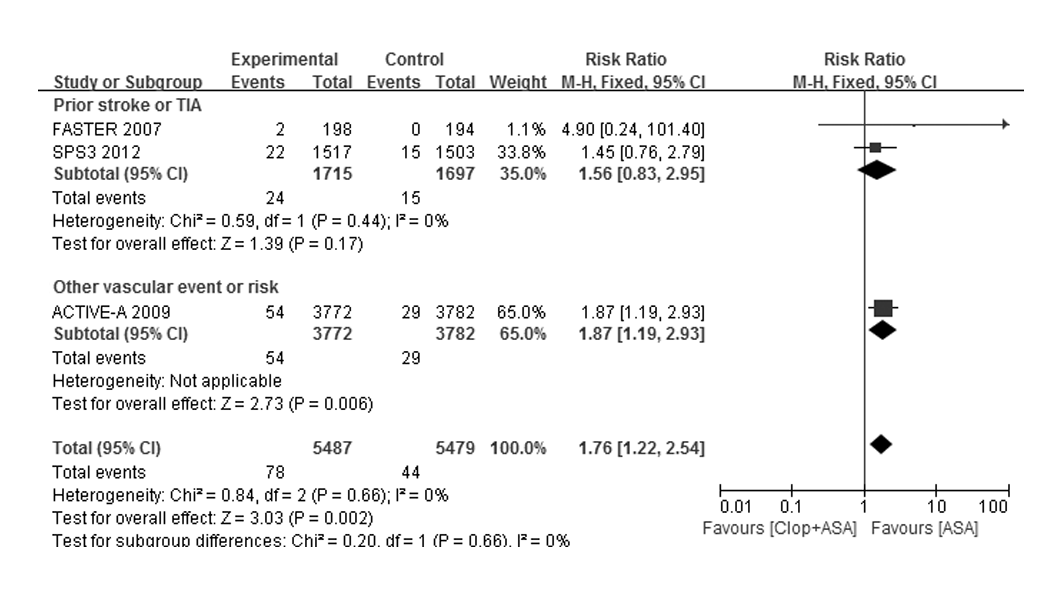

Supplement: Figure S13 — Forest plot of Clop+ASA vs. ASA on intracranial bleeding with long-term treatment. ASA indicates aspirin; CI, confidence interval; Clop, clopidogrel; and M-H, Mantel-Haenszel method. (TIF) [file pone.0104402.s013.tif]

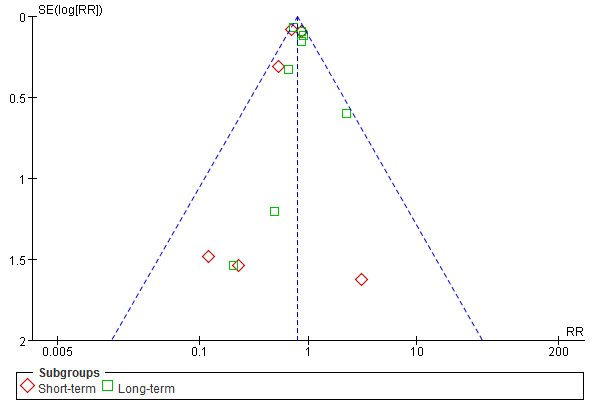

Supplement: Figure S14 — Funnel plot on the outcome of all stroke. RR: relative risk; SE: standard error. (TIF) [file pone.0104402.s014.tif]

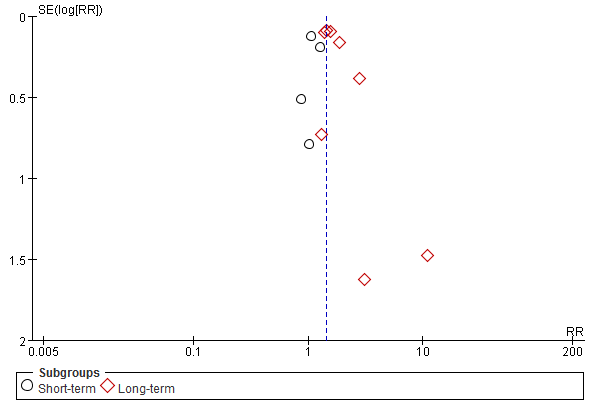

Supplement: Figure S15 — Funnel plot on the outcome of major bleeding. RR: relative risk; SE: standard error. (TIF) [file pone.0104402.s015.tif]

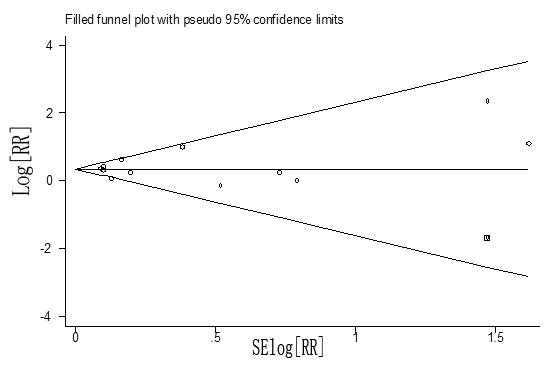

Supplement: Figure S16 — Funnel plot on the outcome of major bleeding after adjustment of publication bias by the “trim and fill” method (by Stata 12.0). RR: relative risk; SE: standard error. (TIF) [file pone.0104402.s016.tif]
