# Supplementary material for: Efficacy and Safety of Adding Clopidogrel to Aspirin on Stroke Prevention among High Vascular Risk Patients: A Meta-Analysis of Randomized Controlled Trials
Source: PLoS One. 2014 Aug 11;9(8):e104402. doi: 10.1371/journal.pone.0104402 (PMC4128803; doi:10.1371/journal.pone.0104402)
Supplement: Table S1 — Supplemental data for baseline characteristics. (DOC) [file pone.0104402.s017.doc]

Table S1: Supplemental data for baseline characteristics

| Study | Nation or Race  (% Exp/Ctrl if given) | BMI(kg/m^2^, mean±SD)  Exp/Ctrl | Medical history—Exp/Ctrl | | | | | Smoker  Exp/Ctrl |
| --- | --- | --- | --- | --- | --- | --- | --- | --- |
|  |  |  | MI | AF | HBP | DM | Hyperlipidemia |  |
| CARESS 2005 | European | NR | 6/10 | NR | 38/31 | 16/18 | 28/32 | NR |
| CLAIR 2010 | Asian | NR | 3/3 | NR | 27/35 | 21/16 | 23/16 | 21/30 |
| COMMIT 2005 | Chinese | NR | 1972/1846 | NR | 9935/9903 | NR | NR | NR |
| CHANCE 2013 | Chinese | 25±2.22/25±2.96 | 43/53 | 48/48 | 1716/1683 | 550/543 | 290/283 | 1116/1105 |
| CLARITY 2005 | White(89.6/89.5) | NR | 159/159 | NR | 750/764 | 289/286 | 564/574 | 887/865 |
| Sun JC, 2010 | Canadian | 28.6±5.2/32.6±4.4 | 23/16 | NR | 34/35 | 18/17 | 33/42 | 21/29 |
| FASTER 2007 | White(93.4/90.2)) | NR | 11/8 | 3/2 | 92/106 | 24/18 | 13/15 | 54/48 |
| Ussia GP 2011 | Italian | NR | 7/4 | 4/6 | 35/31 | 13/8 | NR | NR |
| CURE 2001 | Canadian | NR | 2029/2015 | NR | 3750/3642 | 1405/1435 | NR | 3790/3841 |
| CASCADE 2010 | Canadian | 28.4±4.0/28.4±3.8 | NR | NR | 27/30 | 14/19 | 49/50 | 6/9 |
| CASPAR 2010 | European | 25.6±4.3/25.7±4.2 | NR | NR | 298/298 | 159/162 | 214/208 | 165/155 |
| REAL /ZEST-LATE 2010 | South Korean | NR | 51/45 | NR | 775/765 | 340/364 | 586/584 | 404/431 |
| CHARISMA 2006 | White(80.4/79.9),  Hispanic(9.9/10.7) | NR | 2672/2725 | 298/285 | 5719/5764 | 3304/3252 | 5748/5787 | 5382/5386 |
| SPS3 2012 | White(52/52),Hispanic(31/31),Black(17/17) | NR | NR | NR | 1153/1112 | 531/571 | NR | 303/316 |
| ACTIVE-A 2009 | Global | 28.2±4.9/28.2±4.9 | 525/553 | All | 3217/3210 | 734/728 | NR | NR |

Exp/Ctrl: numbers of patients in experimental group and control group, separately. NR: not reported; BMI: body mass index; TIA: transient ischemic attack; MI: myocardial infarction; AF: Atrial fibrillation; HBP: hypertension; DM: diabetes mellitus.
