# Supplementary material for: Efficacy and Safety of Adding Clopidogrel to Aspirin on Stroke Prevention among High Vascular Risk Patients: A Meta-Analysis of Randomized Controlled Trials
Source: PLoS One. 2014 Aug 11;9(8):e104402. doi: 10.1371/journal.pone.0104402 (PMC4128803; doi:10.1371/journal.pone.0104402)
Supplement: Table S2 — Risk of bias assessment in details. (DOC) [file pone.0104402.s018.doc]

Table S2: Risk of bias assessment in details.

| Study | Random sequence generation | Allocation concealment | Blinding (participants& personnel) | Blinding (outcome assessment) | Incomplete outcome data | Selective reporting | Other bias |
| --- | --- | --- | --- | --- | --- | --- | --- |
| CARESS 2005 | Not reported | By center in balanced blocks of size 4 | Double-blind | Yes | Low risk | Low risk | Unclear |
| CLAIR 2010 | Randomization website | 1:1 in blocks of 4 or 6 (stratified by center) | Open-label^#^ | Yes | Low risk | Low risk | Unclear |
| COMMIT 2005 | Draw lots | Sealed study treatment cases | Open-label^#^ | Yes | Low risk | Low risk | Unclear |
| CHANCE 2013 | Automated system | Numbered medication kit | Double-blind | Yes | Low risk | Low risk | Low risk |
| CLARITY 2005 | Computer | Centrally randomized | Double-blind,open after  angiography | Yes | Low risk | Low risk | Low risk |
| Sun JC, 2010 | Computer | Numbered, pre-prepared bags | Double-blind | Yes | Low risk | Unclear | Unclear |
| FASTER 2007 | In blocks by trial biostatistician | Identical numbered study-treatment kits | Double-blind | Yes | Low risk | Low risk | Unclear |
| Ussia GP 2011 | Not reported | Not reported | Open-label | Yes | Low risk | Unclear | Small sample size |
| CURE 2001 | Computer | Centrally randomized, permuted-block | Double-blind | Yes | Low risk | Low risk | Low risk |
| CASCADE 2010 | Computer | Coordinated by hospital pharmacies | Double-blind | Yes | Low risk | Low risk | Unclear |
| CASPAR 2010 | Pre-established scheme | Interactive voice-  response system | Double-blind | Yes | Low risk | Low risk | Unclear |
| REAL-LATE/ZEST-LATE 2010 | Computer | Stratified on the sites and drugs in the stents | Open-label^#^ | Yes | Low risk | Low risk | Low risk |
| CHARISMA 2006 | Pre-established scheme | interactive voice-response system | Double-blind | Yes | Low risk | Low risk | Low risk |
| SPS3 2012 | Permuted blocks | Centrally randomized, protected from previewing | Double-blind | Yes | Low risk | Low risk | Low risk |
| ACTIVE-A 2009 | Not reported † | Interactive telephone system | Double-blind | Yes | Low risk | Low risk | Low risk |

†: Low risk was assessed considering the reported methods of allocation concealment.

#: Low risk was assessed as open-label brought no substantial bias in these trials.
