# Supplementary material for: Efficacy and Safety of Adding Clopidogrel to Aspirin on Stroke Prevention among High Vascular Risk Patients: A Meta-Analysis of Randomized Controlled Trials
Source: PLoS One. 2014 Aug 11;9(8):e104402. doi: 10.1371/journal.pone.0104402 (PMC4128803; doi:10.1371/journal.pone.0104402)
Supplement: Text S1 — Study protocol. (DOC) [file pone.0104402.s019.doc]

**Meta-analysis of randomized controlled trials to evaluate the efficacy and safety of adding clopidogrel to aspirin on stroke prevention among High Vascular Risk Patients: study protocol**

**Background**

Antiplatelet therapy has been recommended basically for stroke prevention in high vascular risk patients and aspirin has been considered as the first choice though its efficacy seems modest. Adding clopidogrel to aspirin has been promoted for better efficacy while the risk of hemorrhagic complication has attracted eye balls of physicians and neurologists. Whether clopidogrel should be added to aspirin for stroke prevention remains controversial and is only recommended on patients who are assessed as low-risk of bleeding nowadays.

We reviewed the previous meta-analyses [1,2,3,4,5] studying the efficacy of adding clopidogrel to aspirin, but found that the eligible studies had quite different treatment durations of dual antiplatelet therapy, ranging from 7 days to 3.6 years, which may affect the evaluation results of efficacy and safety. However, the reviewers hadn’t put this factor into consideration and hadn’t suggested a suitable duration for combination of clopidogrel and aspirin on stroke prevention. Therefore, we initiate this meta-analysis trying to provide more convincing evidence on the efficacy and safety of the combination of clopidogrel and aspirin on stroke prevention in high vascular risk patients and to suggest a suitable treatment duration for this combination therapy.

**Objectives**

To assess the efficacy and safety of the combination of clopidogrel and aspirin on stroke prevention and to suggest a suitable treatment duration for this combination therapy.

**Methods**

***Inclusion criteria***

1. Human studies without restriction to primary diseases and duration of follow-up.
2. Comparing the combination of clopidogrel and aspirin with aspirin alone;
3. Reporting the incidence of stroke or hemorrhage;
4. Randomized controlled trials;
5. Restricted to English only;
6. Including the report with the most complete data when more than one publication were generated from one study.

***Pre-specified Outcomes***

Primary outcomes of this meta-analysis will be pooled Relative Risks (RRs) for clopidogrel plus aspirin vs. aspirin alone on all stroke (both ischemic and hemorrhagic) and major bleedings requiring blood transfusion. Our secondary outcomes will be pooled RRs for ischemic stroke, hemorrhagic stroke and intracranial bleeding. Trials will be classified into short-term (≤ 1 month) and long-term (≥ 3 months) subgroups according to the treatment duration of clopidogrel plus aspirin.

***Conception of major bleeding***

Major bleeding was evaluated for safety consideration in our meta-analysis. However, hemorrhagic events in the included trials were graded by different sets of criteria-GUSTO criteria, TIMI criteria, CURE criteria, self-defined grades similar to one of those three sets of criteria or without clear definition. Though previous systemic reviews and meta-analyses [1,2,3,4,5] had pooled severe bleeding in GUSTO criteria and major bleeding in other criteria into analysis, we reviewed literature[6,7] and found that different criteria may provide different RRs for evaluating the effect. For more comprehensive consideration, we studied the grading criteria in all of the included trials carefully and finally accepted that major bleeding in our study included the severe and moderate grades in GUSTO criteria, the major and minor grades in TIMI criteria, the major grade in CURE criteria and self-defined major bleeding requiring blood transfusion. Meta-regression would be used to test the heterogeneity of grading criteria if there would be significant evidence of heterogeneity of effect across trials.

***Search Strategy***

Electronic databases including PubMed, EMBase,OVID and the *Cochrane Central Register of Controlled Trials* (up to June 2013) will be searched to identify studies comparing the combination of clopidogrel and aspirin with aspirin alone, restricted to English only. Keywords, PubMed MeSH and free texts search will be combined with the following keywords: clopidogrel, aspirin, Plavix, dual antiplatelet therapy, monotherapy, stroke, bleeding, hemorrhage, hemorrhagic, RCTs, randomized controlled trial.

***Review methods***

1. To remove the duplicate publications and records obviously unrelated to clopdogrel and aspirin.
2. To screen the titles and abstracts of the remaining records and classify them into the following categories:

--relevant: randomized controlled trials on human comparing the combination of clopidogrel and aspirin with aspirin alone;

--possibly relevant: reports fulfil the criteria for the relevant category but not all the required information was provided in the abstract;

--interesting: studies that may lead to relevant articles (e.g. meta-analysis of dual antiplatelet therapy), or any other studies that are interesting from methodological point of view;

--not relevant: not RCT, not in English, not comparing the combination of clopidogrel and aspirin with aspirin alone.

3) To review the full-text of the studies classified as relevant or possibly relevant categories. Possibly relevant articles will be assessed for their potential eligibility and excluded with recorded reason if they couldn’t fulfil the eligibility criteria. Different reports from the same trial will be grouped and the report with the largest sample size will be adopted for meta-analysis while the others will be reserved for potential use. The remaining articles will be assessed for the availability of outcome of interest. In PDF-files, we will do this by searching the following terms: stroke, hemorrhagic, hemorrhage.

4) To scrutinize the reference lists of articles in interesting categories and previous related meta-analyses to reveal additional related articles and assess their eligibility.

***Data extraction***

Data will be extracted independently by 2 investigators. Discrepancies will be resolved by consensus or a third author adjudication. The following data will be abstracted: 1)details of participants’ characteristics including age, sex, body mass index(BMI), race or nation, medical history (previous stroke and TIA, previous myocardial infarction, known atrial fibrillation or flutter, hypertension, diabetes mellitus, hypercholesterolemia), current or previous smoker, primary disease; 2)interventions in each group, including doses and treatment durations of the study drugs; 3) definitions of the pre-specified outcomes; 4) positive events of pre-specified outcomes and total numbers of participants in each group, durations of follow-up, loss of follow-up, intention-to-treat analysis.

***Assessment of risk of bias***

Risk of bias will be assessed independently by 2 investigators. Discrepancies will be resolved by consensus or a third author adjudication. According to the Cochrane collaboration’s tool for assessing risk of bias, we will assess the risk of bias of the included RCTs with the following domains: generation of random sequence; allocation concealment; blinding of participants and personnels; blinding of outcome assessment; incomplete outcome data; selecting reporting; and other potential sources of bias.

***Statistical analysis***

Statistical analysis will be performed independently by 2 investigators. We will consult the statisticians to verify our statistical methods and results, and to resolve the discrepancies during data synthesis. Results of this meta-analysis will be expressed as pooled RRs with 95% confidence intervals (CIs) for dichotomous outcomes. A value of P <0.05 will be considered statistically significant. Heterogeneity across trials will be assessed via a standard Chi square test with significance being set at P<0.10 and also assessed by means of *I*^2^. An *I*^2^ value >50% will be defined as high heterogeneity. Fixed-effect model will be used for statistical analysis when low heterogeneity would be assessed. Random-effect model will be used when there would be high heterogeneity across studies. Subgroup analysis based on treatment duration of clopidogrel plus aspirin will be performed to find out effect of the pre-specified outcomes with different treatment durations. Subgroup analysis based on primary diseases will be performed to evaluate the effect of combination therapy on different populations. Sensitivity analysis will be performed for measuring the effect of included RCTs. Meta-regression will be performed to recognize the sources of heterogeneity if there would be significant evidence of heterogeneity of effect. Funnel plots will be used to screen for publication bias. If the funnel plots present asymmetrical, the “trim and fill” methods will be used to adjust the potential publication bias. Statistical analysis will be performed on Review Manager 5.2 (The Cochrane Collaboration, Oxford, England) and stata 12.0 (StataCorp LP, USA).

**References:**

1. Palacio S, Hart RG, Pearce LA, Anderson DC, Sharma M, et al. (2013) Effect of addition of clopidogrel to aspirin on stroke incidence: Meta-analysis of randomized trials. Int J Stroke.

2. Palacio S, Hart RG, Pearce LA, Benavente OR (2012) Effect of addition of clopidogrel to aspirin on mortality: systematic review of randomized trials. Stroke 43: 2157-2162.

3. Zhou YH, Wei X, Lu J, Ye XF, Wu MJ, et al. (2012) Effects of combined aspirin and clopidogrel therapy on cardiovascular outcomes: a systematic review and meta-analysis. PLoS One 7: e31642.

4. Bowry AD, Brookhart MA, Choudhry NK (2008) Meta-analysis of the efficacy and safety of clopidogrel plus aspirin as compared to antiplatelet monotherapy for the prevention of vascular events. Am J Cardiol 101: 960-966.

5. Geeganage CM, Diener HC, Algra A, Chen C, Topol EJ, et al. (2012) Dual or mono antiplatelet therapy for patients with acute ischemic stroke or transient ischemic attack: systematic review and meta-analysis of randomized controlled trials. Stroke 43: 1058-1066.

6. Yusuf S, Zhao F, Mehta SR, Chrolavicius S, Tognoni G, et al. (2001) Effects of clopidogrel in addition to aspirin in patients with acute coronary syndromes without ST-segment elevation. N Engl J Med 345: 494-502.

7. Rao SV, O'Grady K, Pieper KS, Granger CB, Newby LK, et al. (2006) A comparison of the clinical impact of bleeding measured by two different classifications among patients with acute coronary syndromes. J Am Coll Cardiol 47: 809-816.
